# Practical Questions for Securing Nucleic Acid Synthesis

**DOI:** 10.1089/apb.2023.0028

**Published:** 2024-09-18

**Authors:** Sophie Rose, Tessa Alexanian, Max Langenkamp, Helena Cozzarini, James Diggans

**Affiliations:** ^1^ The Centre for Long-Term Resilience, London, United Kingdom.; ^2^ International Biosecurity and Biosafety Initiative for Science (IBBIS), Geneva, Switzerland.; ^3^ SecureDNA, Zug, Switzerland.; ^4^ Independent, Vienna, Austria.; ^5^ Twist Bioscience, South San Francisco, California, USA.

**Keywords:** biosecurity, synthetic biology, nucleic acid synthesis screening, DNA synthesis, synthetic genomics

## Abstract

**Introduction:**

Affordable and accurate nucleic acid synthesis is foundational to modern biotechnology, but raises security concerns because it facilitates the construction of pathogens and other potentially dangerous biological agents. Nucleic acid synthesis screening can reduce the risk of providing potentially harmful nucleic acids to those without a legitimate use for them. Governments, industry associations, and biosecurity organizations have offered guidance on synthesis screening for a decade, and are now considering how to translate industry best practices into regulatory frameworks.

**Methods:**

A review of existing guidance documents, policy proposals, and other published literature was performed.

**Results:**

We distinguish five categories of practical questions for policymakers: challenges associated with customer screening, sequence screening, the interaction between domestic and global regulations, commercial implications of screening, and finally, challenges associated with benchtop nucleic acid synthesis devices. There are a number of recommendations in public literature that target the implementation of robust customer and sequence screening, several of which have been incorporated into the recent United States White House Executive Order on artificial intelligence. There appears to be fewer solutions proposed to address challenges associated with the global screening landscape, or the commercial implications of screening requirements, and limited discussion on securing the benchtop synthesis landscape.

**Discussion and Conclusion:**

This paper aims at providing a comprehensive resource for policymakers, outlining a set of questions governments, and other stakeholders, must answer when implementing screening requirements to secure nucleic acid synthesis.

## Introduction

Nearly two decades ago, the U.S. National Science Advisory Board for Biosecurity (NSABB)^1^ stated:

It is now feasible to produce synthetic genomes that encode novel and taxonomically unclassified agents with properties equivalent to, or potentially more harmful than, current Select Agents… Biosecurity concerns stem from advances in synthesis technology that make the manipulation and creation of DNA sequences more simple, faster and more accessible.

Gene synthesis costs have since decreased by more than an order of magnitude^2,3^ and DNA has become simpler to manipulate. Biosecurity concerns can be addressed by screening orders for nucleic acids that may encode pathogens or toxins, referred to as “sequences of concern” (SOCs).* Synthesis screening then aims at preventing people without a peaceful, legitimate end-use from purchasing SOCs, making it harder for bad actors to acquire dangerous biological agents.

Governments,^4^ industry associations,^5^ and biosecurity organizations^6^ have offered guidance on synthesis screening but, as of this writing, no countries require nucleic acid orders to be screened before they are fulfilled, despite the recommendation by the NSABB to do so 17 years ago. Implementing screening requirements to secure nucleic acid synthesis screening at a national and global level has proved difficult; this paper will outline the challenges various stakeholders, including national governments, will need to overcome.

### Nucleic Acid Synthesis Screening Workflow

We focus on commercial purchases of nucleic acids from Providers who synthesize and ship orders to Customers. Customers may be scientific End Users or Third-Party Vendors who sell products based on nucleic acids.^†^

Public facing guidance^4,7^ describes best practices for screening as consisting of two parts:

● **Sequence screening** to determine whether the order contains SOCs.
● **Customer screening** to verify the Customers' identity and the scientific legitimacy of their orders.

**Figure 1.**
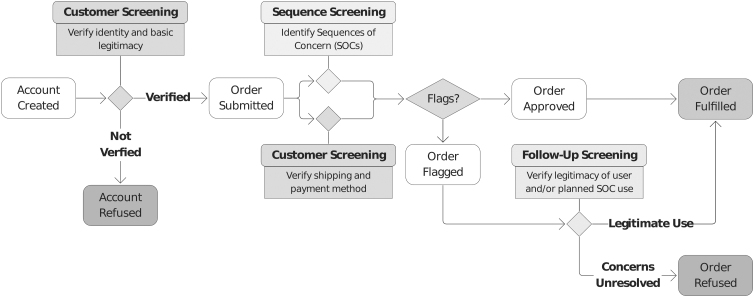
Synthetic nucleic acid purchase workflow, including sequence screening to identify SOCs and customer screening, which occurs when an account is created (verifying identity and basic legitimacy) and when an order is submitted (verifying shipping and payment method). If an order is flagged, follow-up screening verifies the legitimacy of the user and their intended end-use. SOCs, sequences of concern.

The integration of sequence and customer screening practices into the workflow for purchasing synthetic nucleic acids is described in Figure 1.

This workflow differs for benchtop synthesis machines. Customers purchase machines (and often proprietary reagents) from Manufacturers of benchtop equipment, but order fulfillment involves onsite synthesis, rather than shipment of nucleic acids from Providers to Customers.

### Existing Governance Structures

No government currently requires nucleic acid synthesis screening or mandates how it should be performed. Despite this, International Gene Synthesis Consortium (IGSC) members—who represent a majority of the global market—voluntarily screen orders using IGSC's Harmonized Screening Protocol.^5^

This status quo has significant shortcomings. First, the lack of legal mandate means bad actors could order SOCs from companies who do not screen. Second, the voluntary screening paradigm commercially disadvantages companies who do screen, due to the labor costs of the niche expertise required to manually assess flagged orders.^8^ The commercial disadvantage worsens as synthesis costs fall and screening constitutes an increasing fraction of overall order cost.

Rapid advancements at the interface of artificial intelligence (AI) and the life sciences further challenge the status quo, as they have the potential to alter the threat landscape by broadening bioweapon design possibilities.^9,10^

Governments are exploring options for requiring nucleic acid synthesis screening. The White House's *Executive Order on the Safe, Secure, and Trustworthy Development and Use of Artificial Intelligence* (Executive Order on AI) requires a framework for nucleic acid synthesis screening be developed and ties eligibility for federal funding to procurement from Providers in compliance with that framework.^11^

This will build on the recent update to the Screening Framework Guidance by the U.S. Department of Health and Human Services (HHS).^4^ The UK Government's 2023 Biological Security Strategy also references a need to identify a responsible approach to DNA synthesis technology governance.^12^

Successful screening regimes require that governments answer a number of practical questions. Table 1 lists a set of such questions, across five categories: (1) customer screening, (2) sequence screening, (3) the domestic and global landscape, (4) commercial implications, or (5) benchtop synthesis machines.

**Table 1.** Practical questions for governments

***Category***	***Questions***
Customer screening	2.1. How do we verify a customer as a legitimate member of the scientific community? 2.2. How do we verify whether the proposed end-use of SOCs is legitimate? 2.3. How do we account for third parties that order on behalf of End Users? 2.4. How do we ensure nucleic acids are only sent to the approved End User?
Sequence screening	3.1. How do we set standards that ensure adequate screening tool performance? 3.2. What information should be disclosed about SOCs and flagged sequences, and to whom? 3.3. How can we secure dual-use information in sequence screening databases? 3.4. How do we ensure screening is robust to technological advances? 3.5. How do we prevent bad actors from splitting orders to evade screening?
Domestic and global screening landscape	4.1. How should countries structure their nucleic acid synthesis screening regulations? 4.2. How do we design national regulatory regimes that can handle international orders well? 4.3. What can governments do to incentivize screening?
Commercial implications	5.1. How do we mandate screening without undue burden on Providers? 5.2. How do we protect customer information and intellectual property? 5.3. Who is liable if screening fails?
Benchtop synthesis machines	6.1. How can we prevent circumvention of sequence screening on benchtop machines? 6.2. How can we prevent circumvention of customer screening on benchtop machines?

SOCs, sequences of concern.

For each of those categories, we provide a range of recommendations, ranging from existing best practices recommended by public guidance such as the updated HHS Screening Framework Guidance or the U.S. White House Executive Order on AI, and emerging best practices, which require the development of entirely new technical solutions or frameworks, to future proposals. In doing so, we highlight existing solutions, those currently under development and avenues for future work (Table 2).

**Table 2.** Status and source of recommendations

***Recommendation***	***Status***			***Source***		
	***Existing best practice***	***Emerging best practice***	***Future proposals***	***U.S. White House AI EO^11^***	***U.S. HHS Screening Framework Guidance^4^***	***IGSC Harmonised Screening Protocol^5^***
Customer screening						
Develop standards for verifying customer legitimacy		●		**◼**		
Investigate centralized customer verification framework			●			
Encourage preemptive disclosure of customer end-use	●				**◼**	
Develop standards on legitimate end-uses for known SOCs		●				
Incorporate a second approver into end-use verification			●			
Require third parties to conduct equivalent customer screening	●				**◼**	
Investigate individual identifiers for End Users			●			
Record transfers of SOCs		●			**◼**	
Sequence screening						
Develop standards for auditing sequence screening performance		●		**◼**		
Support ongoing synthesis screening platform audits		●		**◼**		
Determine appropriate threat models for informing screening requirements			●	**◼**		
Require secure storage of SOC databases		●		**◼**	**◼**	
Secure the digital infrastructure surrounding nucleic acid synthesis			●		**◼**	
Consider screening informed by function in addition to taxonomic lists		●			**◼**	
Allow rapid updates to the screening database			●			
Require small screening windows	●				**◼**	
Build infrastructure for secure sharing of flags between Providers			●			
Require alignment of small sequences within and across customer orders	●				**◼**	
Domestic and global						
Specify comprehensive workflows for screening		●		**◼**		
Identify an entity and process for reporting orders	●			**◼**		
Facilitate international coordination on regulation			●			
Mandate customer and sequence screening			●			
Require government-funded research use screened nucleic acids		●		**◼**		
Commercial						
Develop specialized genome databases			●			
Government-led development of infrastructure and resources			●			
Develop free or low-cost screening tools			●			
Provide financial support to Providers who screen			●			
Rigorous cybersecurity to protect customer information		●			**◼**	
Clarify liability and how it is affected by screening practices			●			
Benchtop synthesis						
Require Manufacturers to screen sequences before synthesis		●			**◼**	**◼**
Encourage adoption of a security standard and audits for benchtop manufacturers	●				**◼**	
Require digital authorization for machine use			●			
Require verification for reagent sales	●				**◼**	

● Circles indicate the status of the recommendation.

◼ Squares indicate the initial source of a recommendation.

We categorize each recommendation presented into one of three groups: (1) “Existing best practice” recommendations are feasible to implement, but not yet universal, and are included in prominent nucleic acid screening policies as described by the source column; (2) “Emerging best practice” recommendations are supported by prominent screening policies or some current screening tools, but require more development before universal implementation is feasible; (3) “Future proposals” denotes recommendations that do not form part of any of these documents or tools and rather highlight areas of future work.

AI, artificial intelligence; IGSC, International Gene Synthesis Consortium.

## Customer Screening

Customer screening measures involve verifying the Customer's identity (“Know Your Customer” or KYC) and other basic checks (e.g., many Providers will not ship to a P.O. box or residential address). If an order is flagged, follow-up customer screening determines whether the Customer, and their proposed end-use, are scientifically legitimate^4,5^ (Figure 1). KYC measures are a common, although not fully standardized, best practice in other sectors, such as financial services, where they help to enforce laws around anti-money laundering and countering terrorism.^13^ But they have not yet been standardized in the synthesis industry.

### How Do We Verify a Customer as a Legitimate Member of the Scientific Community?

There is no established consensus for what constitutes a “legitimate” Customer (e.g., Do scientists need related publications to order an SOC? Is a company registration sufficient for verifying a diagnostics startup?). Though the *Companion Guide for Implementation of the Screening Framework Guidance*^7^ includes *Use Cases on Verifying Legitimacy*, it does not define who is considered legitimate.

#### Recommendations

##### Develop standards for verifying customer legitimacy

National governments should work with stakeholders to define the scope of legitimacy in this context. This could include verification of the customer's intended use for the ordered material and access to appropriate containment facilities (where appropriate), customer's affiliation with an institution and history of published papers in related domains to their proposed work, or evidence that they are part of an institution that can offer biosafety review and experimental oversight for work related to requested SOCs. The U.S. OSTP was recently tasked with developing such a standard by the Executive Order on AI.^11^

*Investigate the creation of a centralized, national Customer verification framework*, which could be used across the life sciences.^14^

### How Do We Verify Whether the Proposed End-Use of SOCs is Legitimate?

Despite verification of a Customers' proposed end-use being recommended by the IGSC and Screening Framework Guidance, there are no definitions for what constitutes legitimate end-use. Currently, Providers determine ad-hoc whether flagged SOCs are needed for the proposed end-use and, in some cases, make a risk-benefit assessment of the end-use. Both require substantial niche expertise, which may be financially burdensome, especially for smaller companies.

#### Recommendations

##### Encourage preemptive disclosure of proposed end-use by customers

Customers should be encouraged to preemptively provide information alongside orders containing SOCs to assist the provider in verifying their legitimacy.^4^ Some synthesis screening tools allow Customers to submit their research purpose and biosafety officer contact information.^15^

##### Develop standards on legitimate end-uses for known SOCs

Screening Framework Guidance recommends evaluating whether a Customer's proposed end-use is legitimate,^4^ but it is unclear whether legitimacy refers only to the customer's profile, or should also include a risk-benefit assessment of the customer's proposed end-use. Providers would benefit from guidance on how to verify whether flagged SOCs are necessary for a proposed end-use. Standards for legitimacy could be informed by the U.S. National Institutes of Health (NIH)'s Dual-Use Research guidance, and rule out end-uses that are likely to result in certain, concerning experimental effects.

##### Incorporate a second approver into end-use verification

To guard against illegitimate use of legitimate Customer accounts, Providers could require a second approver to verify legitimacy before fulfilling orders. Where possible, this should be an institutional biosafety officer providing evidence of institutional review and approval; Customers without a designated biosafety contact will need to nominate a second approver, and criteria for who can serve as a second approver must be developed.

### How Do We Account for Third Parties That Order on Behalf of End-Users?

Third-party vendors who order sequences on behalf of End Users can complicate customer screening. For instance, vendors may be hesitant to disclose their client's identity or share proprietary customer information.^16^ The Screening Framework Guidance assigns the same responsibilities to third-party vendors as it does to Providers of synthetic nucleic acids.^4^

#### Recommendations

##### Require third-party vendors to conduct equivalent customer screening

Following recent guidance, third-party vendors should document to whom they are distributing a product and conduct follow-up screening for orders containing SOCs.^4^ Sequence screening by third-party vendors is unnecessary if this was already performed by the Provider.

### How Do We Ensure Nucleic Acids are Only Sent to the Approved End User?

Common practices in academia and industry make it challenging to ensure synthetic nucleic acids are only used by approved End Users. Purchasing accounts are often shared across research groups, and researchers commonly share genetic material without explicit Material Transfer Agreements (MTA).^17^

#### Recommendations

##### Investigate individual identifiers for end users

Some experts recommend incorporating requirements to ascertain the identity of End Users, such as individual PINs, similar to best practices in drug prescribing,^16,18^ though no authority currently exists to provide such identifiers.

##### Record some transfers of SOCs

Recent guidance^4^ suggests that SOCs should only be transferred from a Customer to an additional End User after the user's legitimacy is verified. However, in some jurisdictions, transfers of whole agents that contain SOCs (e.g., in the United States, sequences known to confer pathogenicity from a pathogen not on the Federal Select Agent Program list) are not recorded; record-keeping standards should specify when transfers should be recorded and what information to capture.

## Sequence Screening

Sequence screening involves comparing the nucleic acid sequences in orders to known SOCs. Current approaches use a taxonomic “best match” that relies on sequence homology to determine whether sequences likely originate from controlled biological agents. “Best match” approaches can be augmented by curated SOC databases and algorithmic prediction of functions of concern.^19,20^

The genomes of regulated organisms and emerging pathogens are usually shared in public sequence databases, including open-access databases or those available to registered users. However, these databases are not optimized for identifying taxonomic “best matches”^21^ and often do not annotate sequence by function. As such, synthesis Providers and providers of screening tools typically develop their own proprietary sequence databases and algorithms for identifying SOCs.

There are alternative possible structures for *centralized* curated SOC databases, including a government-housed database or a shared Provider database (such as the IGSC's database), each with unique benefits and limitations (see What Information Should Be Disclosed About SOCs and Flagged Sequences, and To Whom? section, How Can We Secure Dual-Use Information in Sequence Screening Databases? section and How Do We Ensure Screening is Robust to Technological Advances? section).

### How Do We Set Standards That Ensure Adequate Screening Tool Performance?

Several sequence screening tools are available (ThreatSeq, FAST-NA, SeqScreen, Aclid)^21–24^ or in beta testing (Common Mechanism, SecureDNA).^25,26^ These tools, as well as Providers' internal screening tools, employ different SOC databases and algorithms for identifying sequences that match their databases. A benchmark would provide metrics to compare platforms and verify compliance with future regulations.^16,27^ The U.S. National Institute of Standards and Technology (NIST) was tasked with developing such a framework in the Executive Order on AI.^11^

#### Recommendations

##### Create a government standard for auditing sequence screening performance

Existing taxonomic databases cannot be directly used as a standard for screening performance, as they include numerous inaccurately or ambiguously labeled sequences.^21^

##### Support regular synthesis screening platform audits

Currently, IGSC members' screening capabilities are audited only upon joining. Regular audits would better ensure that screening is sustained.^28^ Audits would benefit from a framework for conducting structured evaluation of screening platforms.^11^

### What Information Should Be Disclosed About SOCs and Flagged Sequences, and To Whom?

Genomes of regulated pathogens are well known, and newly discovered pathogen genomes are typically shared publicly for public health purposes. Therefore, some experts argue there is limited value in securing such sequences. However, curated databases of pathogen sequences could make it easier to create AI tools that could be misused to design viruses for intentional release.^29^

This question is among those where we identified the least consensus about appropriate solutions; as such, we primarily present a series of further questions for discussion:

1. Will Governments request screening tools to reference a centralized database to ensure newly discovered SOCs are added? How might this be done if Governments lack the technical resources to curate SOCs for a centralized database?
2. The principle of least privilege in computer security suggests that access (to the SOC database) be restricted to those who strictly require it (database maintainers). Accordingly, maintainers of a centralized database may not wish to share the full contents of the SOCs with others due to dual-use concerns. How do we deal with concerns about dual-use information in terms of the contents of the SOC database?
3. When novel dual-use sequences are identified, such as the mousepox mutation that conferred immune escape,^30^ it may be appropriate to incorporate those into screening databases before publicly disclosing. How might we ensure this?
4. Providers may not wish to use a third-party screening tool that requires them to send Customers' sequences to a remote server due to intellectual property concerns. Can this be fully alleviated through certain encryption methods?^31^

#### Discussion

Decisions must also be made about what information should be disclosed about the outcome of sequence screening, particularly in cases of suspected misuse. Sufficiently suspicious orders may warrant notifying legal authorities (e.g., repeated attempts to order SOCs with slight modifications and no scientific justification). The IGSC Harmonized Screening Protocol includes commitments to establish relationships with law enforcement.^5^

However, to protect Customer intellectual property and privacy, Providers may wish to provide only select details. This difficulty is compounded by the fact that providing details to Customers about flagged sequences could help bad actors to prioritize among SOCs or reverse-engineer and subvert screening.

#### Recommendation

##### Form consensus on the appropriate threat models for nucleic acid screening

Various stakeholders should discuss and come to consensus on the appropriate threat model to use for nucleic acid screening. This threat model should be informed by discussion with experts and empirical data where possible. If consensus can be reached, many of the aforementioned questions can be addressed more clearly.

### How Can We Secure Dual-Use Information in Sequence Screening Databases?

As discussed, there is disagreement about how to best structure and manage access to SOC databases. Some experts feel there are circumstances in which it is appropriate to limit access to the full contents of a database (see What Information Should Be Disclosed About SOCs and Flagged Sequences, and To Whom? section). Best practices for security and access controls of SOC databases have not yet emerged, though the Executive Order on AI calls for their development.^11^

#### Recommendations

##### Require secure storage of SOC databases

Any database of deliberately non-public SOCs will require security to prevent unauthorized access or exfiltration. Best practices in computer security indicate that sensitive information should not be stored in hardware that is physically accessible to end users, such as benchtop synthesis devices, and should instead be stored remotely.^32^

##### Secure the digital infrastructure surrounding nucleic acid synthesis

Bad actors may attempt cyberattacks to steal the contents of a private SOC or customer sequence databases, or infiltrate Provider equipment to synthesize an SOC.^33^ Cybersecurity measures could be strengthened via periodic red teaming efforts, minimum security requirements, and bug bounty programs.

### How Do We Ensure Screening is Robust to Technological Advances?

Many sequence screening protocols are based on measuring similarity to the genomes of organisms on control lists,^20^ but this may not capture underlying functions of concern. In addition, taxonomic control lists are limited in their robustness to deliberate obfuscation of known threats, and may not capture newly discovered pathogens or novel agents designed with AI tools.^9,10^

#### Recommendations

##### Consider using screening informed by function in addition to taxonomic lists

There are a range of screening approaches informed by the function of the underlying sequence. The most simple account for recoding amino acids (i.e., where a different nucleic acid sequence generates an identical protein). The most complex involve machine-learning algorithms to predict sequence functions, though tools for functional prediction are nascent.

They also require an SOC database annotated according to function, such as the Functions of Sequences of Concern database, which are currently scarce and small in size, as well as resource-intensive to generate.^34^ Thus, widespread integration of functional screening is not yet feasible. Justifying voluntary functional sequence screening may also be challenging, given existing regulations are based on taxonomic lists.

##### Allow rapid updates to sequence databases used for screening

SOC databases should be updated as new SOCs are discovered. This could be achieved by requiring screening tools to reference a centralized database and defining a protocol for adding new SOCs to the database, along with a clear standard for which sequences qualify as SOCs. Benchtop DNA synthesizers (see the Benchtop Synthesis Machines section) will have to be adapted to ensure that updates propagate to benchtop screening.

### How Do We Prevent Bad Actors from Splitting Orders to Evade Screening?

Screening could be evaded by splitting orders into smaller parts, then distributing them between multiple Providers or across a prolonged time period. We observe analogous circumvention of security in the financial industry (e.g., money laundering via use of multiple financial institutions) and methamphetamine production (e.g., purchase of smaller quantities of methamphetamine precursor, pseudoephedrine, from multiple pharmacies).

#### Recommendations

##### Require small screening windows

Having small windows for sequence screening may reduce risk from split orders. If the screening window is 50 nucleotides, as suggested in the Screening Framework Guidance, it becomes difficult for a bad actor to synthesize a complete SOC given the sheer number of assembly steps required.

##### Create infrastructure for Providers to securely share information about flagged orders with other Providers

The IGSC Harmonized Screening Protocol encourages members to notify other IGSC members on identifying potentially problematic orders/Customers,^5^ though this mechanism is rarely used.^16^ Providers may be uncomfortable sharing sensitive customer information, but information-sharing infrastructure could be designed that does not leak Customer sequences, using approaches such as homomorphic encryption,^35^ or pseudo-alignment.^27^

##### Require alignment of small sequences within and across a Customer's orders

The Screening Framework Guidance suggests “screening all sequences ordered by an individual Customer, using a short sequence alignment software package.”^4^ This may facilitate the detection of spaced acquisition attempts if Providers detect orders for short sequences that over time could be assembled into SOCs, but does not address one Customer ordering from several different Providers.^27^

## Domestic and Global Screening Landscape

The global synthetic biology market revenue for oligonucleotides and synthetic DNA was estimated at 4.76 U.S. billion dollars in 2022 and is set to grow,^36^ incentivizing Providers to participate in the international market. As different governments consider approaches to governing nucleic acid synthesis, Providers may be burdened by discordant national screening requirements. In addition to the need to tailor national screening regulations to their unique domestic regulations and structure, governments may benefit from introducing legal requirements or financial incentives to compel screening.

### How Should Countries Structure Their Nucleic Acid Synthesis Screening Regulations?

To regulate nucleic acid synthesis, countries must assign responsibilities for implementation and oversight based on the structure of their governmental organizations. This will have particular implications for law enforcement, national security, non-proliferation, and export control bodies.

#### Recommendations

##### Specify comprehensive workflows for nucleic acid synthesis screening

Providers need explicit guidance on how to comply with new regulations. National governments should ensure that these workflows interact with existing law enforcement, national security, and export control activities in this space.^37^

##### Designate an entity for handling reports of suspicious orders and establish procedures for suspicious order reporting

For example, in the United States, this role is currently fulfilled by FBI Field Office Weapons of Mass Destruction (WMD) Coordinators.^4^

### How Do We Design National Regulatory Regimes That Can Handle International Orders Well?

Many major Providers fulfill international orders and may face the burden of complying with various, discordant national regulations to serve the international market.^15^ Differences in domestic regulations will also introduce vulnerabilities if bad actors can order from Providers in areas without screening requirements.

#### Recommendation

##### Facilitate international coordination on screening regulations and standards underlying them

Countries should coordinate when designing regulatory regimes and screening standards to harmonize regulations and reduce the burden on Providers operating internationally.

#### Discussion

The development of standards for synthesis screening by organizations such as the International Organization for Standardization (ISO) could also help set a baseline that Providers operating internationally can adhere to.^38^

### What Can Governments Do to Incentivize Screening?

The nucleic acid synthesis industry, especially through the IGSC,^15^ has been at the forefront of developing best practices to mitigate the risks associated with their technology. However, it is costly to implement, scale, and maintain effective screening practices, which creates a commercial disadvantage that serves as a disincentive for robust screening.

#### Recommendations

In addition to establishing government standards for evaluating screening tool performance (see Require Secure Storage of SOC Databases Recommendation)—a necessary precursor to verifying compliance with screening requirements—governments could:

##### Mandate customer and sequence screening

Legally requiring all domestic synthesis companies to screen according to government standards would increase screening rates and overall quality, and may also help shift international norms. However, mandatory screening nationally may lead Customers to source nucleic acids from cheaper foreign Providers that are not required to screen.^39^

##### Require that all government-funded research sources nucleic acid from companies that are compliant with government standards.^40^

In many countries, a significant fraction of synthetic nucleic acids are purchased for government-funded research^36^ and governments can incentivize screening by requiring this customer base to purchase from Providers that screen.^15^ This measure could address concerns about outsourcing and was recently included in the Executive Order on AI.^11^

## Commercial Implications

Synthetic nucleic acids are a commercial product essential to the bioeconomy Safeguarding innovation will be critical for bolstering national bioeconomies, a priority reflected in documents such as the White House's Executive Order on *Bold Goals for U.S. Biotechnology and Biomanufacturing.*^41^ Given the falling price of synthesis per nucleotide and increasing labor costs, it is becoming more difficult for companies who screen to remain cost competitive with companies who do not.^27^ Mandating screening or broadening its scope could exacerbate this dynamic by increasing the number of flagged orders. A robust and sustainable regulatory regime must consider the commercial implications of regulations.

### How Do We Mandate Screening Without Undue Burden on Providers?

Implementing screening can be costly for Providers. Approximately 5% of synthetic DNA orders are flagged and reviewing one flag can take several hours,^40^ as it requires (1) expert evaluation to determine whether a flag is a true positive and (2) verifying Customer legitimacy. Larger companies often operate with greater profit margins and may be better positioned to absorb additional resource burdens associated with a mandatory screening regime, whereas smaller companies may become less competitive after shifting costs to their Customers.^42^

#### Recommendations. This burden on Providers could be alleviated through

##### Development specialized genome databases for use by screening tools

Developing genetic databases that clearly scope what constitutes an SOC (e.g., via the removal of “housekeeping genes” with high expression levels, conserved sequences, and known non-pathogenic function)^40^ could reduce the false positive rate.

##### Government-led development of infrastructure and resources

Government-led development of screening infrastructure and resources, such as SOC databases and AI models trained to identify SOCs based on those databases, could alleviate some of the burden faced by Providers, and ensure a standardized industry approach.

##### Development of free or low-cost screening tools

Supporting the development of ultimately free or low-cost tools may partially reduce the in-house resources companies need to perform customer or sequence screening.^42,43^

##### Providing financial offsets to Providers who screen

Financial support to offset the costs of compliance can be used to encourage adoption of best practice and help Providers remain competitive in the international marketplace.^28^

#### Discussion

Financial offsets could aim to prioritize smaller Providers: for instance, providing offsets on a sliding scale relative to Provider size. Regulatory “sandboxes” (i.e., contained regulatory environments that allow emerging technologies or approaches to be tested) have previously been recommended as a mechanism to assess regulatory impact on innovation across different-sized companies.^44^ These have been implemented in financial services sectors globally,^45^ though there has been limited exploration of sandboxes for nucleic acid synthesis screening.^37^

### How Do We Protect Customer Information and Intellectual Property?

Synthesis orders can contain valuable intellectual property. Screening tools, data storage, and processes for passing information on to law enforcement will need to be designed to protect customer data and prevent corporate espionage.

#### Recommendation

##### Providers should implement high-quality security and cybersecurity measures (e.g., ISO 27001 or NIST 800-53) to protect their Customers' information

Resources, such as SOC reporting databases for industry or commercial screening platforms, should be designed with privacy in mind.^4^

### Who Is Liable If Screening Fails?

With the exception of international orders captured by export controls, it is unclear who would be liable for a harmful biological event originating with synthetic nucleic acids. Free or low-cost screening tools (see Governments Should Clarify Liability and How It is Affected by Screening Practices Recommendation) may lose value if liability concerns push Providers to develop their own in-house tools for verifying screening results.

#### Recommendation

##### Governments should clarify liability and how it is affected by screening practices

Numerous publications have recommended the exploration of potential mechanisms for financial^46,47^ or legal liability^16,42^ to encourage compliance, but progress appears limited. Governments should explore potential liabilities faced by Providers who supply bad actors with SOCs and liability shields for Providers shown to be in compliance with screening best practices.

## Benchtop Synthesis Machines

The majority of synthetic nucleic acids are currently produced by centralized Providers. However, new biosecurity and governance challenges are emerging as benchtop synthesis devices become increasingly affordable and accessible.

### How Can We Prevent Circumvention of Sequence Screening on Benchtop Machines?

Currently, synthetic DNA capabilities are centralized: Providers are able to screen orders, often in-house, before their fulfillment. In contrast, benchtop synthesizers are designed so that Customers can “print” DNA in their own labs. The hardware is directly accessible to Customers, vastly increasing the potential for misuse by unauthorized modification of hardware or software.

#### Recommendations

##### Require manufacturers to screen sequences before synthesis

Devices could report sequences to a secure cloud-based screening tool or using screening software embedded in the device firmware.^48^ This should be at least as rigorous as centralized synthesis screening. Screening capabilities should be retained regardless of a device's internet access and devices detecting tampering should refuse to synthesize.

##### Encourage adoption of a security standard and audits for benchtop manufacturers^4^

The standard should draw on best practices from the computer security industry, including: authenticating users, securing hardware interfaces, and adding basic anti-tamper and secure networking measures. This standard should describe mechanisms for securely updating device firmware, to secure against malware and other cyber threats, and screening software, to ensure screening keeps pace as new hazards are discovered. Periodic audits should be done to ensure a minimal standard of security is adhered to (similar to e.g., SOC2 for financial vendors^49^).

### How Can We Prevent Circumvention of Customer Screening on Benchtop Machines?

Benchtop devices are significantly more prone to customer screening circumvention, due to accessibility (devices are accessible to a wide range of individuals^48^) and ease of device or sequence resale (malicious or careless actors may exploit cybersecurity vulnerabilities to remove software restrictions and then resell devices or sequences they produced; see, for instance, the indirect sale of devices to embargoed countries reported by benchtop assembly manufacturer Telesis^50^).

#### Recommendations

##### Require digital authorization for machine use

Each individual End-User should have unique credentials, ideally following best practices for multi-factor authentication. By default, machine access should be time-limited, with users logged out after each session,^4^ to limit inadvertent sharing of credentials.

##### Require verification for reagent sales

Benchtop devices require regular refilling of reagents, many of which are proprietary and must be purchased directly from Providers. Ensuring reagents are sold to registered End-Users (and not a different individual, or third-party reseller) and may help limit third-party use.^4^

## Conclusion

The existing framework for voluntary screening is inadequate, and set to worsen as synthetic biology and AI advance.

Yet, there is reason for cautious optimism. Since the NSABB recommended nucleic acid synthesis screening in 2006, significant progress has been made: best practices have emerged, free sequence screening software is available, and advancements in AI may improve sequence interpretation. Collectively, these developments create favorable conditions for government action to implement screening requirements.

Implementing those requirements requires answering several questions. This paper identifies eighteen specific questions, summarized in Table 1, spanning five categories: (1) customer screening, (2) sequence screening, (3) the domestic and global regulatory landscape, (4) commercial implications, or (5) benchtop synthesis machines.

To meet these challenges, we offer 33 recommendations, which will require a collaborative effort across various stakeholder groups to successfully implement. Table 2 categorizes these recommendations according to existing and emerging best practice, as well as highlighting those that are avenues for future work. Table 3 provides a summary of these recommendations, and delineates whether governments, Providers and Manufacturers, Customers, scientific institutions, and synthesis screening providers are responsible for each.

**Table 3.** Stakeholder contributions to recommendations

***Recommendation***	***Responsible actor 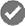 Indicates primary responsibility***				
	***Governments***	***Providers and manufacturers***	***Customers***	***Scientific institutions***	***Synthesis screening providers***
Customer screening					
Develop standards for verifying customer legitimacy	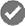	●	●	●	●
Investigate centralized Customer verification framework	●	●			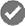
Encourage preemptive disclosure of customer end-use	●	●			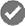
Develop standards on legitimate end-uses for known SOCs	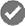	●		●	
Incorporate a second approver into end-use verification	●			●	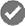
Require third parties to conduct equivalent customer screening	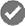	●	●		
Investigate individual identifiers for End Users	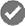	●			●
Record some transfers of SOCs		●	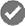	●	
Sequence screening					
Develop standards for auditing sequence screening performance	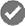	●		●	●
Support ongoing synthesis screening platform audits	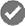	●			●
Determine appropriate threat models for informing screening requirements	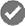	●		●	●
Require secure storage of SOC databases	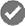	●			●
Secure the digital infrastructure surrounding nucleic acid synthesis	●	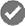			●
Consider screening informed by function in addition to taxonomic lists	●	●			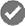
Allow rapid updates to the screening database	●	●			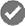
Require small screening windows	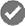				●
Build infrastructure for secure sharing of flags between Providers	●	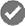			●
Require alignment of small sequences within and across customer orders	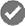	●			●
Domestic and global					
Specify comprehensive workflows for screening	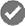				
Identify an entity and process for reporting orders	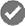				
Facilitate international coordination on regulation	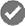				
Mandate customer and sequence screening	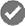				
Require government-funded research use screened nucleic acids	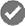				
Commercial					
Develop specialized genome databases	●				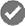
Government-led development of infrastructure and resources	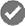				
Develop free or low-cost screening tools	●				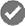
Provide financial support to Providers who screen	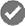			●	
Rigorous cybersecurity to protect customer information		●			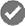
Clarify liability and how it is affected by screening practices	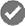				
Benchtop synthesis					
Require Manufacturers to screen sequences before synthesis	●	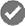			
Encourage adoption of a security standard and audits for benchtop manufacturers	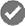	●		●	●
Require digital authorization for machine use	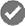	●		●	
Require verification for reagent sales	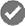	●			

We refer to Providers and Manufacturers and Customers as defined by the Screening Framework Guidance.^4^ “Scientific Institutions” refers to organizations where research involving nucleic acids may occur, such as pharmaceutical or academic research groups. We use “Synthesis Screening Providers” to refer to organizations that provide tools for nucleic acid screening. We label the actor best positioned to lead a successful implementation as the “primarily responsible” stakeholder (denoted by the gray check mark) and indicate with a dot those actors who are well positioned to assist as “partially responsible.” To ensure successful implementation, we suggest ultimate accountability rests with the “primarily responsible” actor.

We call on these actors to implement these recommendations and refine their approach to securing nucleic acid synthesis screening.

## Notes

^*^
SOCs are defined in the U.S. Screening Framework Guidance for Providers and Users of Synthetic Nucleic Acids (Screening Framework Guidance)^4^ as “A nucleotide sequence that is a ‘Best Match’ to a sequence of federally regulated agents … except when the sequence is also found in an unregulated organism or toxin.” The Guidance recommends extending this to incorporate “sequences known to contribute to pathogenicity or toxicity, even when not derived from or encoding regulated biological agents” as soon as it is practical to do so.

^†^
Definitions of Providers, Customers, End Users, Third-Party Vendors, and Manufacturers follow those given in the Screening Framework Guidance.

## References

[1] National Science Advisory Board. Addressing Biosecurity Concerns Related to the Synthesis of Select Agents. Washington, DC; 2006.

[2] Carlson R. Carlson Curves—Synthesis. 2016. Available from: http://www.synthesis.cc/synthesis/category/Carlson+Curves [Last accessed: November 13, 2023].

[3] Twist Bioscience. High Quality Gene Synthesis-Twist Bioscience. 2023. Available from: https://web.archive.org/web/20230419180640/https://www.twistbioscience.com/products/genes?tab=overview [Last accessed: November 28, 2023].

[4] U.S. Department of Human Health Services. Screening Framework Guidance for Providers and Users of Synthetic Nucleic Acids (October 2023).

[5] International Gene Synthesis Consortium. IGSC Harmonized Screening Protocol. 2017. Available from: https://genesynthesisconsortium.org/wp-content/uploads/IGSCHarmonizedProtocol11-21-17.pdf [Last accessed: December 12, 2023].

[6] Nuclear Threat Initiative. Preventing the Misuse of DNA Synthesis Technology. 2023. Available from: http://www.nti.org/about/programs-projects/project/preventing-the-misuse-of-dna-synthesis-technology/ [Last accessed: November 15, 2023].

[7] U.S. Department of Human Health Services. Companion Guide to Assist in Implementing the Recommendations of the Screening Framework Guidance for Providers and Users of Synthetic Nucleic Acids. n.d.

[8] HHS Screening Framework Guidance for Providers and Users of Synthetic Oligonucleotides, Summary of Updates in Response to Public Comments Received in 2020. Available from: https://aspr.hhs.gov/legal/syndna/Documents/Summary-Updates-508.pdf [Last accessed: December 12, 2023].

[9] The Centre for Long-Term Resilience. Examining Risks at the Intersection of AI and Bio. 2023. Available from: https://www.longtermresilience.org/post/report-launch-examining-risks-at-the-intersection-of-ai-and-bio [Last accessed: November 8, 2023].

[10] Sandbrink JB. Artificial Intelligence and Biological Misuse: Differentiating Risks of Language Models and Biological Design Tools. 2023; doi: 10.48550/arXiv.2306.13952.

[11] White House. Executive Order on the Safe, Secure, and Trustworthy Development and Use of Artificial Intelligence. 2023. Available from: https://www.whitehouse.gov/briefing-room/presidential-actions/2023/10/30/executive-order-on-the-safe-secure-and-trustworthy-development-and-use-of-artificial-intelligence/ [Last accessed: November 8, 2023].

[12] UK HM Government. UK Biological Security Strategy. 2023. Available from: https://assets.publishing.service.gov.uk/government/uploads/system/uploads/attachment_data/file/1173779/UK_Biological_Security_Strategy.pdf [Last accessed: November 15, 2023].

[13] Arner DW, Zetzsche DA, Buckley RP, et al. The identity challenge in finance: From analogue identity to digitized identification to digital KYC utilities. Eur Bus Organ Law Rev 2019;20(1):55–80; doi: 10.1007/s40804-019-00135-1

[14] Nuclear Threat Initiative. The Convergence of Artificial Intelligence and the Life Sciences. 2023. Available from: https://www.nti.org/analysis/articles/the-convergence-of-artificial-intelligence-and-the-life-sciences/ [Last accessed: November 8, 2023].

[15] Aclid Industry Insights Newsletter. Aclid Announces $3.3M Funding Round to Automate Biosecurity. 2023.

[16] Engineering Biology Research Consortium. Security Screening in Synthetic DNA Synthesis: Recommendations for Updated Federal Guidance. 2022. Available from: https://ebrc.org/wp-content/uploads/2022/04/EBRC-2022-Security-Screening-in-Synthetic-DNA-Synthesis.pdf [Last accessed: November 30, 2023].

[17] Kahl L, Molloy J, Patron N, et al. Opening options for material transfer. Nat Biotechnol 2018;36(10):923–927; doi: 10.1038/nbt.4263 30307930 PMC6871013

[18] Williams B, Kane R. Preventing the Misuse of DNA Synthesis. Institute for Progress. 2023. Available from: https://ifp.org/preventing-the-misuse-of-dna-synthesis/ [Last accessed: December 12, 2023].

[19] Godbold GD, Hewitt FC, Kappell AD, et al. Improved understanding of biorisk for research involving microbial modification using annotated sequences of concern. Front Bioeng Biotechnol 2023;11:1124100. 37180048 10.3389/fbioe.2023.1124100 PMC10167326

[20] Millett P, Alexanian T, Brink KR, et al. Beyond biosecurity by taxonomic lists: Lessons, challenges, and opportunities. Health Secur 2023; In Press; doi: 10.1089/hs.2022.0109 PMC10733751 37856148

[21] Beal J, Clore A, Manthey J. Studying pathogens degrades BLAST-based pathogen identification. Sci Rep 2023;13(1):5390; doi: 10.1038/s41598-023-32481-z 37012314 PMC10068195

[22] Battelle. Threat SEQ™ DNA Screening Web Service|Battelle Service Solution. n.d. Available from: https://www.battelle.org/markets/health/chemical-and-biological-countermeasures/biosecurity-pandemic-preparedness/threatseq [Last accessed: November 9, 2023].

[23] Balaji A, Kille B, Kappell AD, et al. SeqScreen: accurate and sensitive functional screening of pathogenic sequences via ensemble learning. Genome Biol 2022;23(1):133; doi: 10.1186/s13059-022-02695-x 35725628 PMC9208262

[24] Aclid. Aclid-About. n.d. Available from: https://www.aclid.bio/about [Last accessed: November 25, 2023].

[25] Secure DNA. Secure DNA-Fast, Free, and Accurate DNA Synthesis Screening. n.d. Available from: https://securedna.org/ [Last accessed: November 9, 2023].

[26] Nuclear Threat Initiative. Common Mechanism to Prevent Illicit Gene Synthesis. 2019. Available from: https://www.nti.org/analysis/articles/common-mechanism-prevent-illicit-gene-synthesis/ [Last accessed: November 25, 2023].

[27] Diggans J, Leproust E. Next steps for access to safe, secure DNA synthesis. Front Bioeng Biotechnol 2019;7:86. 31069221 10.3389/fbioe.2019.00086 PMC6491669

[28] Nuclear Threat Initiative. Biosecurity Innovation and Risk Reduction Initiative (BIRRI). 2023. Available from: https://www.nti.org/about/programs-projects/project/fostering-biosecurity-innovation-and-risk-reduction/ [Last accessed: February 27, 2024].

[29] Sandbrink J, Ahuja J, Swett J, et al. Mitigating Biosecurity Challenges of Wildlife Virus Discovery. SSRN. 2022. Available from: https://papers.ssrn.com/sol3/papers.cfm?abstract_id=4035760 [Last accessed: December 12, 2023].

[30] Lewis G, Millett P, Sandberg A, et al. Information hazards in biotechnology. Risk Anal 2019;39(5):975–981. 30419157 10.1111/risa.13235 PMC6519142

[31] Computer Security Division ITL. Hash Functions|CSRC|CSRC. 2017. Available from: https://csrc.nist.gov/projects/hash-functions [Last accessed: November 8, 2023].

[32] Scarfone KA, Jansen W, Tracy M. Guide to General Server Security. 0 ed. National Institute of Standards and Technology: Gaithersburg, MD; 2008.

[33] Puzis R, Farbiash D, Brodt O, et al. Increased cyber-biosecurity for DNA synthesis. Nat Biotechnol 2020;38(12):1379–1381; doi: 10.1038/s41587-020-00761-y 33247280

[34] Godbold GD, Kappell AD, LeSassier DS, et al. Categorizing sequences of concern by function to better assess mechanisms of microbial pathogenesis. Infect Immun 2022;90(5):e00334-21; doi: 10.1128/iai.00334-21 34780277 PMC9119117

[35] Esvelt KM. Inoculating science against potential pandemics and information hazards. PLOS Pathog 2018;14(10):e1007286; doi: 10.1371/journal.ppat.1007286 30286188 PMC6171951

[36] Precedence Research. Synthetic Biology Market Size to Hit USD 116.04BnBy2032. n.d. Available from: https://www.precedenceresearch.com/synthetic-biology-market [Last accessed: November8, 2023].

[37] The Centre for Long-Term Resilience. Overcoming Challenges with Synthetic Nucleic Acid Screening Implementation. 2023. Available from: https://www.longtermresilience.org/post/report-launch-overcoming-challenges-with-synthetic-nucleic-acid-screening-implementation [Last accessed: February 27, 2024].

[38] 14:00-17:00.ISO/FDIS20688-2. n.d. Available from: https://www.iso.org/standard/75852.html [Last accessed: November 14, 2023].

[39] Institute for Progress. Preventing the Misuse of DNA Synthesis. n.d. Available from: https://ifp.org/preventing-the-misuse-of-dna-synthesis/ [Last accessed: November 15, 2023].

[40] Carter SR, Friedman R. DNA Synthesis and Biosecurity: Lessons Learned and Options for the Future. J Craig Venter Institute. Available from: https://www.jcvi.org/research/dna-synthesis-and-biosecurity-lessons-learned-and-options-future

[41] White House. Bold Goals for U.S. Biotechnology and Biomanufacturing: Harnessing Research and Development to Further Societal Goals. 2023. Accessible from: https://www.whitehouse.gov/wp-content/uploads/2023/03/Bold-Goals-for-U.S.-Biotechnology-and-Biomanufacturing-Harnessing-Research-and-Development-To-Further-Societal-Goals-FINAL.pdf [Last accessed: November 30, 2023].

[42] Maurer SM, Fischer M, Schwer H, et al. Making Commercial Biology Safer: What the Gene Synthesis Industry Has Learned About Screening Customers and Orders. 2009. Available from: https://www.semanticscholar.org/paper/Making-Commercial-Biology-Safer%3A-What-the-Gene-Has-Maurer-Fischer/11562b7d22c1750264ed2d8208f9b702dd6c34ffu [Last accessed: November 30, 2023].

[43] Hoffmann SA, Diggans J, Densmore D, et al. Safety by design: Biosafety and biosecurity in the age of synthetic genomics. iScience 2023;26(3):106165; doi: 10.1016/j.isci.2023.106165 36895643 PMC9988571

[44] Regulatory Horizons Council. Report on Genetic Technologies. 2021. Available from: https://assets.publishing.service.gov.uk/government/uploads/system/uploads/attachment_data/file/1089198/regulatory_horizons_council_report_on_genetic_technologies_july_2022.pdf. [Last accessed: November 30, 2023].

[45] World Bank. Global Experiences from Regulatory Sandboxes. 2020. Available from: https://documents1.worldbank.org/curated/en/912001605241080935/pdf/Global-Experiences-from-Regulatory-Sandboxes.pdf [Last accessed: February 27, 2024].

[46] Appleton E, Millett P. Technical Aspects of Biosecurity: Screening Guidance, Attribution, and Traceability. In: Emerging Threats of Synthetic Biology and Biotechnology: Addressing Security and Resilience Issues. (Trump BD, Florin M-V, Perkins E, et al. eds) Springer: Dordrecht, DE; 2021. 36122110

[47] Nuclear Threat Initiative. Biosecurity Innovation and Risk Reduction: A Global Framework for Accessible, Safe and Secure DNA Synthesis. World Economic Forum. 2020. Available from: https://www3.weforum.org/docs/WEF_Biosecurity_Innovation_Risk_Reduction.pdf [Last accessed: November 30, 2023].

[48] Nuclear Threat Initiative. Benchtop DNA Synthesis Devices: Capabilities, Biosecurity Implications, and Governance. 2023. Available from: https://www.nti.org/analysis/articles/benchtop-dna-synthesis-devices-capabilities-biosecurity-implications-and-governance/ [Last accessed: November 8, 2023].

[49] Institute of Internal Auditors. SOC 2 Breakdown: A Five-Part Guide to Understanding the Service Organization Controls 2 Report and Its Benefits - Document - Gale Academic OneFile. n.d. Available from: https://go.gale.com/ps/i.do?id=GALE%7CA281683691&sid=googleScholar&v=2.1&it=r&linkaccess=abs&issn=00205745&p=AONE&sw=w&userGroupName=mlin_oweb&isGeoAuthType=true&aty=geo [Last accessed: November 25, 2023].

[50] Telesis. Form 10-Q. Financial Report. 2022. Available from: https://ir.telesisbio.com/static-files/62a7d286-0bf3-4af7-beed-9e27744090e9 [Last accessed: November 30, 2023].

